# Phenotypic and Genotypic Characterization of *Enterobacteriaceae* Producing Oxacillinase-48–Like Carbapenemases, United States

**DOI:** 10.3201/eid2404.171377

**Published:** 2018-04

**Authors:** Joseph D. Lutgring, Wenming Zhu, Tom J.B. de Man, Johannetsy J. Avillan, Karen F. Anderson, David R. Lonsway, Lori A. Rowe, Dhwani Batra, J. Kamile Rasheed, Brandi M. Limbago

**Affiliations:** Emory University School of Medicine, Atlanta, Georgia, USA (J.D. Lutgring);; Centers for Disease Control and Prevention, Atlanta (J.D. Lutgring, W. Zhu, T.J.B. de Man, J.J. Avillan, K.F. Anderson, D.R. Lonsway, L.A. Rowe, D. Batra, J.K. Rasheed, B.M. Limbago)

**Keywords:** phenotypic characterization, genotypic characterization, oxacillinase-48–like carbapenemases, OXA-48, beta-lactamases, antimicrobial resistance, Enterobacteriaceae, bacteria, anti-bacterial agents, United States

## Abstract

Oxacillinase (OXA)–48–like carbapenemases remain relatively uncommon in the United States. We performed phenotypic and genotypic characterization of 30 *Enterobacteriaceae* producing OXA-48–like carbapenemases that were recovered from patients during 2010–2014. Isolates were collected from 12 states and not associated with outbreaks, although we could not exclude limited local transmission. The alleles β-lactamase OXA-181 (*bla*_OXA-181_) (43%), *bla*_OXA-232_ (33%), and *bla*_OXA-48_ (23%) were found. All isolates were resistant to ertapenem and showed positive results for the ertapenem and meropenem modified Hodge test and the modified carbapenem inactivation method; 73% showed a positive result for the Carba Nordmann–Poirel test. Whole-genome sequencing identified extended-spectrum β-lactamase genes in 93% of isolates. In all *bla*_OXA-232_ isolates, the gene was on a ColKP3 plasmid. A total of 12 of 13 isolates harboring *bla*_OXA-181_ contained the insertion sequence ΔIS*Ecp1*. In all isolates with *bla*_OXA-48_, the gene was located on a TN*1999* transposon; these isolates also carried IncL/M plasmids.

The prevalence of carbapenem-resistant *Enterobacteriaceae* (CRE) has been increasing in the United States since 2000 (*1**,**2*). This finding is problematic because treatment options for CRE infection are limited, and these infections are associated with a higher mortality rate than are infections with carbapenem-susceptible *Enterobacteriaceae* (*3*). *Enterobacteriaceae* might be resistant to carbapenems by a variety of mechanisms, the most concerning of which is production of carbapenemases (*4*). Although the *Klebsiella pneumoniae* carbapenemase is the most common carbapenemase reported in the United States, there have been reports of several other carbapenemases including the metallo-β-lactamases and, more recently, oxacillinase (OXA)–48–like carbapenemases (*1**,**5**–**10*).

OXA-48 is a member of the ambler class D β-lactamase family, first described in a *K. pneumoniae* isolate from Turkey in 2004 (*11*). The OXA-48 enzyme hydrolyzes penicillins efficiently, carbapenems slowly, and extended-spectrum cephalosporins poorly; it is not inhibited by tazobactam, sulbactam, or clavulanic acid (*12*). Since the initial report, OXA-48 has established reservoirs in Turkey, the Middle East, countries in North Africa, and throughout Europe (*12*). These reservoirs have been reported in multiple *Enterobacteriaceae* species in addition to *K. pneumoniae*, including *Citrobacter freundii*, *Enterobacter cloacae*, *Escherichia coli*, *K. oxytoca*, *Serratia marcescens*, and *Providencia rettgeri* (*12*). In addition to OXA-48, several variants with similar enzymatic profiles have been described, including OXA-162, -181, -204, -232, -244, -245, -370, -436, -438, and -484; each variant differs from OXA-48 by only a few amino acids (*12**–**16*). Other variants that do not hydrolyze carbapenems have also been described, including OXA-163, -247, and -405 (*13**,**17**,**18*).

The first description of isolates with β-lactamase OXA-48–like (*bla*_OXA-48_−like) genes in the United States was from a surveillance study in 2013, which incidentally reported 2 *K. pneumoniae* isolates (*6*). This description was followed shortly afterward by a report of 2 clinical *K. pneumoniae* isolates with *bla*_OXA-48_−like genes in patients from 1 institution in Virginia who had traveled internationally (*7*). More recently, CRE with *bla*_OXA-232_ genes have been isolated in the United States (*8*). The Centers for Disease Control and Prevention (CDC) has collected multiple isolates harboring *bla*_OXA-48_−like genes from patients in the United States (*19*). We report the genotypic and phenotypic characterization of those isolates.

## Materials and Methods

### Collection of Isolates

Isolates are submitted to CDC for antimicrobial susceptibility testing (AST) for many reasons, including outbreak response, AST confirmation, and surveillance studies. Surveillance studies include the Multi-Site Gram-Negative Surveillance Initiative, which is part of the Emerging Infections Program, and the Sentinel Study (*5*,*20*). All *Enterobacteriaceae* isolates received for AST at CDC during June 1, 2010–October 31, 2012, with reduced susceptibility to carbapenems (MIC >1 μg/mL for any carbapenem), a positive modified Hodge test result, and a PCR-negative result for *bla K. pneumoniae* carbapenemase were retrospectively screened for *bla*_OXA-48_−like genes (n = 115). During November 1, 2012–September 30, 2014, all *Enterobacteriaceae* received at CDC were routinely tested for *bla*_OXA-48_−like genes by real-time PCR (n = 1,399). Submitting institutions were characterized by state and US Department of Health and Human Services (HHS) region (https://www.hhs.gov/ash/about-ash/regional-offices/index.html).

### Phenotypic Characterization of Isolates

We performed reference broth microdilution AST on all isolates by using in-house prepared frozen panels that included carbapenems, cephalosporins, aztreonam, penicillins, quinolones, trimethoprim/sulfamethoxazole, aminoglycosides, chloramphenicol, tetracyclines, tigecycline, polymyxin B, and colistin (*21*,*22*). The modified Hodge test, Carba Nordmann–Poirel test, and the modified carbapenem inactivation method (mCIM) were performed on all *bla*_OXA-48_−like isolates according to Clinical and Laboratory Standards Institute guidelines (*22*). We confirmed species identification by using the Biotyper 3.1 MALDI System (Bruker Daltronics, Billerica, MA, USA).

### Genotypic Characterization of Isolates

The PCR for *bla*_OXA-48_−like genes was developed at CDC and detects *bla*_OXA-48_, *bla*_OXA-162_, *bla*_OXA-163_, *bla*_OXA-181_, *bla*_OXA-204_, *bla*_OXA-232_, *bla*_OXA-244_, *bla*_OXA-245_, *bla*_OXA-247_, *bla*_OXA-370_, *bla*_OXA-405_, *bla*_OXA-438_, *bla*_OXA-484_, and *bla*_OXA-505_ by using 2 sets of *bla*_OXA-48_−like primers/probes and a bacterial 16S rRNA gene as an endogenous control for lysate validation and PCR amplification (Table 1). We extracted DNA by using the thermal/sodium hydroxide method for preparation of bacterial cell lysates (*23*). Cycling conditions were a 3-min enzyme activation step at 95°C, followed by 40 cycles for 3 s at 95°C, and a final step for 30 s at 60°C (*24*).We characterized all isolates positive for *bla*_OXA-48_−like genes by using whole-genome sequencing (WGS). We extracted DNA by using the Maxwell 16 Cell Low Elution Volume DNA Purification Kit (Promega, Madison, WI, USA) and fragmented input genomic DNA (gDNA) with an absorbance ratio of 1.8–2.0 to ≈800 bp by an using an ultrasonic fragmentation system (Covaris, Woburn, MA, USA). We prepared libraries by using the Ovation Ultralow DR Multiplex System 1–96 Kit (Nugen Technologies, Inc., San Carlos, CA, USA), then multiplexed, and sequenced with MiSeq V2.0 (Illumina, San Diego, CA, USA). We filtered raw Illumina sequencing reads for quality (average >Q20) and discarded trimmed reads >50 bp from the dataset by using SolexaQA version 3.1 (*25*). We then assembled clean reads into contigs by using SPAdes version 3.1.0 and 4 k-mer sizes (k = 41, 79, 85, and 97) (*26*). Afterward, we mapped trimmed reads back to each assembled genome by using the Burrows-Wheeler Alignment tool for minor contig error correction (*27*).

**Table 1:** Sequences of primers and probes used for identification of *Enterobacteriaceae* isolates with β-lactamase OXA-48−like carbapenemases, United States*

Primers and probes	Sequence, 5′→3′
16S rRNA, forward primer	TGG AGC ATG TGG TTT AAT TCG A
16S rRNA, reverse primer	TGC GGG ACT TAA CCC AAC A
16S rRNA, probe (CY5)	CY5-CA CGA GCT GAC GAC ARC CAT GCA-BHQ
OXA-48, forward 180	ACG GGC GAA CCA AGC AT
OXA-48, reverse 239	GCG ATC AAG CTA TTG GGA ATT T
OXA-48, probe 199	FAM-TT ACC CGC ATC TAC C-BHQ
OXA-48, forward 722	TGC CCA CAT CGG ATG GTT
OXA-48, reverse 781	CCT GTT TGA GCA CTT CTT TTG TGA
OXA-48, probe 741	AG GGC TGC GCC AAG
OXA-48 F1	ATG CGT GTR TTA GCC TTA TC
OXA-48 R1	CTA KGG AAT WAT YTT YTC CTG

*OXA, oxacillinase.

We randomly selected *K. pneumoniae* isolates 1, 11, and 23, encoding *bla*_OXA-181_, *bla*_OXA-232_, and *bla*_OXA-48_, respectively, as internal reference strains and sequenced them by using Single Molecule Real-Time Technology (Pacific Biosciences, Menlo Park, CA, USA) in addition to Illumina sequencing (Table 2). We extracted and purified gDNA by using the MasterPure Complete DNA and RNA Kit (Epicenter, Madison, WI, USA), according to the manufacturer’s recommended protocol. We generated 10-kb libraries by using the SMRTbell Template Prep Kit 1.0 (Pacific Biosciences) and sequenced libraries by using C4v2 Chemistry on the RSII Instrument (Pacific Biosciences). We assembled data by using Hierarchical Genome-Assembly Process version 3.0 (Pacific Biosciences) and generated clean consensus sequences by using Quiver (*28*).

**Table 2:** Phenotypic and genotypic characterization of *Enterobacteriaceae* harboring β-lactamase OXA-48−like carbapenemase, United States*

Isolate no.	Species	Year	Source	HHS region†	MHT MEM	mCIM	Carba NP	ST	OXA allele	NDM allele	ESBLs
1	*Klebsiella pneumoniae*	2010	Urine	9	+	+	+	ST34	181	None	CTX-M-15
2	*K. pneumoniae*	2011	Urine	9	+	+	+	ST34	181	None	CTX-M-15
3	*K. pneumoniae*	2011	Urine	9	+	+	+	ST34	181	None	CTX-M-15
4	*K. pneumoniae*	2011	Urine	9	+	+	+	ST34	181	None	CTX-M-15
5	*K. pneumoniae*	2011	Respiratory	9	+	+	+	ST34	181	None	CTX-M-15
6	*K. ozaenae*	2011	Respiratory	9	+	+	+	None	181	None	CTX-M-15
7	*K. pneumoniae*	2011	Wound	10	+	+	+	ST14	232	None	CTX-M-715
8	*K. pneumoniae*	2012	Peritoneal fluid	3	+	+	–	ST43	181	None	CTX-M-15
9	*K. pneumoniae*	2012	Urine	5	+	+	+	ST14	232	1	CTX-M-15
10	*K. pneumoniae*	2012	Rectal swab	5	+	+	+	ST14	232	1	CTX-M-15
11	*K. pneumoniae*	2013	Urine	3	+	+	+	ST14	232	1	CTX-M-15
12	*K. pneumoniae*	2013	Respiratory	3	+	+	+	ST14	232	1	CTX-M-15
13	*K. pneumoniae*	2013	Respiratory	5	+	+	Ind	ST147	181	None	CTX-M-15
14	*Enterobacter aerogenes*	2013	Peritoneal fluid	3	+	+	+	None	48	None	None
15	*K. pneumoniae*	2013	Urine	6	+	+	–	ST16	232	None	CTX-M-15
16	*K. pneumoniae*	2013	Urine	6	+	+	–	ST16	232	None	CTX-M-15
17	*K. pneumoniae*	2013	Urine	6	+	+	Ind	ST16	232	None	CTX-M-15
18	*K. pneumoniae*	2013	Urine	5	+	+	+	ST14	232	None	CTX-M-15
19	*K. pneumoniae*	2013	Respiratory	5	+	+	Ind	ST43	181	None	CTX-M-15
20	*K. pneumoniae*	2013	Respiratory	5	+	+	+	ST43	181	None	CTX-M-15
21	*K. pneumoniae*	2013	Urine	5	+	+	+	ST15	48	None	CTX-M-15
22	*K. pneumoniae*	2014	Respiratory	1	+	+	+	ST437	181	5	CTX-M-15, SHV-12
23	*K. pneumoniae*	2014	Respiratory	5	+	+	+	ST14	48	None	CTX-M-14b, CTX-M-15
24	*Escherichia coli*	2014	Urine	2	+	+	–	None	181	None	None
25	*K. pneumoniae*	2014	Urine	2	+	+	+	ST36	48	None	None
26	*K. pneumoniae*	2013	Urine	9	+	+	+	ST34	181	None	CTX-M-15
27	*K. pneumoniae*	2013	Urine	9	+	+	Ind	ST34	181	None	CTX-M-15
28	*K. pneumoniae*	2014	Respiratory	4	+	+	+	ST101	48	None	CTX-M-15
29	*K. pneumoniae*	2014	Respiratory	4	+	+	+	ST101	48	None	CTX-M-15
30	*K. pneumoniae*	2014	Unknown	4	+	+	+	ST101	48	None	CTX-M-15

*ESBLs, extended-spectrum β-lactamases; HHS, Health and Human Services; Ind, indeterminate; mCIM, modified carbapenem inactivation method; MEM, meropenem; MHT, modified Hodge test; NDM, New Delhi metallo-β-lactamase gene; NP, Nordmann–Poirel; OXA, oxacillinase; ST, sequence type (by multilocus sequence typing); –, negative; +, positive. †HHS regions: 1, Connecticut, Maine, Massachusetts, New Hampshire, Rhode Island, Vermont; 2, New Jersey, New York; 3, Delaware, District of Columbia, Maryland, Pennsylvania, Virginia, West Virginia; 4, Alabama, Florida, Georgia, Kentucky, Mississippi, North Carolina, South Carolina, Tennessee; 5, Illinois, Indiana, Michigan, Minnesota, Ohio, Wisconsin; 6, Arkansas, Louisiana, New Mexico, Oklahoma, Texas; 7, Iowa, Kansas, Missouri, Nebraska; 8, Colorado, Montana, North Dakota, South Dakota, Utah, Wyoming; 9, Arizona, California, Hawaii, Nevada; 10, Alaska, Idaho, Oregon, Washington.

We deposited all raw sequencing reads, Pacific Biosciences assemblies, and MIC results in GenBank under BioProject PRJNA296771. We determined multilocus sequence types for each specimen by mapping clean Illumina reads to allele sequences (http://www.pubmlst.org) by using SRST2 software (Illumina) (*29*). We described antimicrobial resistance genotype profiles from assembled Illumina and Pacific Biosciences contigs by using SSTAR V1.0 (*30*) in combination with the ARG-ANNOT (*31*) and ResFinder (*32*) repositories.

We used the PlasmidFinder database (http://www.genomicepidemiology.org/) to detect plasmid replicon sequences among Illumina and Pacific Biosciences contigs to estimate the plasmid composition of each isolate (*33*). In addition, we predicted insertion sequences that might be associated with spread of antimicrobial resistance genes by using ISfinder (*34*). For isolates with *bla*_OXA-48_, we estimated the copy number of IS*1R* insertion sequences for determining Tn*1999* variants by using blastn (https://blast.ncbi.nlm.nih.gov/Blast.cgi) and SPAdes K-mer coverage output (*26**,**34**–**36*). The clonality of our plasmids was also assessed, as was the location of *bla*_OXA-48_−like genes (Technical Appendix). Because of a cluster of isolates from 1 state in this study, a phylogenetic tree and single-nucleotide polymorphism (SNP) tree matrix were produced by using RAxML version 8 (*37*) (Technical Appendix).

### Transformation Experiments

We randomly transformed 10 selected isolates (3 with *bla*_OXA-48_, 4 with *bla*_OXA-181_, and 3 with *bla*_OXA-232_) for transformation experiments to better characterize plasmids harboring *bla*_OXA-48_−like genes. We subcultured parent isolates on trypticase soy agar containing 5% sheep blood, placed them in 50 mL of tryptic soy broth containing ertapenem (1 μg/mL), and incubated them overnight at 35°C. We extracted plasmid DNA by using Plasmid Midi Kits (QIAGEN, Valencia, CA, USA), according to the manufacturer’s protocol. We digested intact plasmid DNA and gDNA with *Hin*dIII (New England Biolabs, Ipswich, MA, USA) and separated this DNA by electrophoresis on a 0.9% agarose gel.

We transformed 500 ng of plasmid DNA from each isolate into *E. coli* DH10B cells (Invitrogen, Carlsbad, CA, USA) by electroporation and incubated at 35°C for 2 h. Potential transformants were plated on Luria–Bertani agar containing ertapenem (1 μg/mL) and incubated overnight at 35°C. Four colonies from each transformant plate were screened for *bla*_OXA-48_−like genes by using PCR. Transformant plasmid DNA was digested and separated by gel electrophoresis along with digested parent plasmid DNA to ensure that transformant plasmids were also present in parental cells.

We characterized confirmed transformants by using AST, the modified Hodge test, and WGS with MiSeq V2.0 (Illumina), as described previously. Trimmed reads from transformants were mapped to the genome sequence of *E. coli* K12, substrain DH10B (GenBank accession no. NC_010473.1), by using Bowtie 2 software (*38**,**39*). Unmapped reads were extracted by using bam2fastq (https://gsl.hudsonalpha.org/information/software/bam2fastq) and were considered to represent plasmid DNA harboring *bla*_OXA-48_−like genes (https://gsl.hudsonalpha.org/information/software/bam2fastq). We subsequently assembled these unmapped reads by using SPAdes software and screened generated contigs for antimicrobial drug resistance genes by using SSTAR V1.0 and for plasmid replicon sequences by using the PlasmidFinder database (*26**,**30**,**33*).

## Results

### Epidemiology of Isolates

We included all 30 US isolates in our collection that were positive for a *bla*_OXA-48−_like carbapenemase gene in this study. Isolates were submitted from patients in 12 states representing 8 HHS regions: one from region 1, two from region 2, four from region 3, three from region 4, eight from region 5, three from region 6, eight from region 9, and one from region 10. *K. pneumoniae* predominated (n = 27, 90%), although single isolates of *K. ozaenae*, *Enterobacter aerogenes*, and *E. coli* (n = 1 each, 3%) were also found. Isolates were collected from a variety of sources: urine (n = 15, 50%), respiratory samples (n = 10, 33%), peritoneal fluids (n = 2, 7%), wounds (n = 1, 3%), rectal swab specimens (n = 1, 3%), and unknown sources (n = 1, 3%) (Table 2).

### Phenotypic Characterization of Isolates

All submitted isolates with a *bla*_OXA-48_−like carbapenemase gene showed resistance to ertapenem and all penicillins tested (including those with β-lactamase inhibitors). Most showed intermediate resistance or resistance to imipenem (n = 30, 100%), meropenem (n = 28, 93%), doripenem (n = 28, 93%), ceftriaxone (n = 29, 97%), ceftazidime (n = 27, 90%), and cefepime (n = 28, 93%). In addition, all isolates had a colistin MIC <2 μg/mL (Table 3). Results for the ertapenem modified Hodge test, meropenem modified Hodge test, and mCIM were positive for all isolates harboring *bla*_OXA-48_−like genes. The Carba Nordmann–Poirel test result was positive for 73% of isolates, indeterminate in 13%, and negative in 13% (Table 2).

**Table 3:** MIC results for *Enterobacteriaceae* harboring β-lactamase oxacillinase-48–like carbapenemases, United States*

Isolate no.	Species	Drug, MIC, μg/mL											
		ETP	MEM	IMP	DOR	TZP	CRO	CAZ	FEP	CIP	COL	TIG	AMK
1	*Klebsiella pneumoniae*	>4	4	4	4	>128	>32	>32	>32	>8	<0.25	2	4
2	*K. pneumoniae*	>8	4	2	4	>128	>32	>128	>32	>8	<0.5	2	>64
3	*K. pneumoniae*	>8	4	2	4	>128	>32	>128	>32	>8	1	1	>64
4	*K. pneumoniae*	8	2	4	2	>128	>32	>128	>32	>8	0.5	<0.5	>64
5	*K. pneumoniae*	8	2	2	2	>128	>32	>128	>32	>8	0.5	<0.5	>64
6	*K. ozaenae*	>8	4	4	4	>128	>32	>128	>32	>8	0.5	1	>64
7	*K. pneumoniae*	>8	>8	4	>8	>128	>32	>128	>32	>8	1	4	32
8	*K. pneumoniae*	>8	8	4	8	>128	>32	>128	>32	>8	0.5	1	16
9	*K. pneumoniae*	>8	>8	>64	>8	>128	>32	>128	>32	>8	0.5	2	>64
10	*K. pneumoniae*	>8	>8	64	>8	>128	>32	>128	>32	>8	1	2	>64
11	*K. pneumoniae*	>8	>8	64	>8	>128	>32	>128	>32	>8	0.5	2	>64
12	*K. pneumoniae*	>8	>8	>64	>8	>128	>32	>128	>32	>8	0.5	2	>64
13	*K. pneumoniae*	>8	>8	4	8	>128	>32	128	>32	>8	0.5	4	>64
14	*Enterobacter aerogenes*	2	2	4	2	>128	<1	<1	1	<0.25	0.5	<0.5	<1
15	*K. pneumoniae*	>8	>8	8	>8	>128	>32	>128	>32	>8	0.5	4	>64
16	*K. pneumoniae*	>8	>8	8	>8	>128	>32	>128	>32	>8	0.5	4	>64
17	*K. pneumoniae*	>8	>8	2	8	>128	>32	>128	>32	>8	0.5	4	>64
18	*K. pneumoniae*	>8	>8	64	>8	>128	>32	>128	>32	>8	0.5	2	>64
19	*K. pneumoniae*	>8	>8	8	>8	>128	>32	>128	>32	>8	2	1	<1
20	*K. pneumoniae*	>8	>8	8	>8	>128	>32	>128	>32	>8	2	1	<1
21	*K. pneumoniae*	2	0.25	2	0.5	>128	>32	64	>32	>8	0.5	1	>64
22	*K. pneumoniae*	>8	>8	32	>8	>128	>32	>128	>32	>8	0.5	4	>64
23	*K. pneumoniae*	>8	4	4	4	>128	>32	128	>32	>8	0.5	4	64
24	*Escherichia coli*	4	0.5	2	0.5	>128	8	2	8	>8	0.5	<0.5	2
25	*K. pneumoniae*	>8	8	4	8	>128	2	<1	2	<0.25	1	<0.5	<1
26	*K. pneumoniae*	>8	8	4	8	>128	>32	>128	>32	>8	0.5	1	>64
27	*K. pneumoniae*	>8	2	2	2	>128	>32	128	>32	>8	0.5	4	<1
28	*K. pneumoniae*	>8	>8	4	>8	>128	>32	>128	>32	>8	1	1	8
29	*K. pneumoniae*	>8	>8	32	>8	>128	>32	>128	>32	>8	0.5	<0.5	8
30	*K. pneumoniae*	>8	>8	8	>8	>128	>32	>128	>32	>8	0.5	1	8

*Not all drugs tested are listed. *bla*, β-lactamase; AMK, amikacin; CAZ, ceftazidime; CIP, ciprofloxacin; COL, colistin; CRO, ceftriaxone; DOR, doripenem; ETP, ertapenem; FEP, cefepime; IMP, imipenem; MEM, meropenem; TIG, tigecycline; TZP, piperacillin/tazobactam.

### Transformation Experiments

We purified plasmid DNA from 10 isolates (3 with *bla*_OXA-48_, 4 with *bla*_OXA-181_, and 3 with *bla*_OXA-232_) for transformation into *E. coli* DH10B. Transformants were obtained for each preparation from strains harboring *bla*_OXA-48_ and *bla*_OXA-232_, as confirmed by PCR and phenotypic and genotypic characterization of each transformant (Table 4). Transformation was unsuccessful for all DNA preparations from strains with *bla*_OXA-181_ (isolates 1, 2, 26, and 27).

**Table 4:** Plasmid transformation of *Enterobacteriaceae* producing OXA-48–like carbapenemases, United States*

Isolate no.†	MIC, μg/mL					MHT MEM	OXA allele	Plasmid replicon type	ESBL CTX-M
	ETP	MEM	CRO	TZP	AMK				
R	<0.12	<0.12	<1	<4	<1	–			
7	>8	>8	>32	>128	32	+	232	ColKP3, IncR, IncFIB(pQIL)	15
7T	>8	4	<1	>128	<1	+	232	ColKP3	
9	>8	>8	>32	>128	>64	+	232	ColKP3, IncR, IncHI1B, IncFIB(K)	15
9T	>8	8	<1	>128	<1	+	232	ColKP3	
11	>8	>8	>32	>128	>64	+	232	ColKP3, IncR, IncHI1B, IncFIB(K)	15
11T	>8	8	≤1	>128	<1	+	232	ColKP3	
23	>8	4	>32	>128	64	+	48	IncL/M	14b, 15
23T	4	1	>32	>128	16	+	48	IncL/M	14b
25	>8	8	2	>128	<1	+	48	IncL/M	
25T	>8	8	<1	>128	<1	+	48	IncL/M	
28	>8	>8	>32	>128	8	+	48	IncL/M, IncR, ColRNAI	
28T	>8	8	<1	128	<1	+	48	IncL/M	

*AMK, amikacin; CRO, ceftriaxone; ESBL, extended-spectrum β-lactamase; ETP, ertapenem; MEM, meropenem; MHT, modified Hodge test; OXA, oxacillinase; R, recipient strain before transformation (*Escherichia coli* DH10B); T, transformant; TZP, piperacillin/tazobactam; –, negative; +, positive. †Isolates 1, 2, 26, and 27 did not have any transformants.

When we compared transformants with parent strains, most of which harbored multiple plasmids and numerous resistance genes, transformants were confirmed to carry only 1 plasmid and typically showed greater susceptibility to extended-spectrum cephalosporins but retained resistance to >1 carbapenem. As confirmed by WGS, we found that ESBL genes were not typically present on the same plasmid as *bla*_OXA-48_−like genes; only 1 transformant (23T) carried a plasmid harboring *bla*_CTX-M-14b_ on the IncL/M plasmid carrying *bla*_OXA-48_. Similar to the parent strain, strain 23T showed increased MICs to cephalosporins and carbapenems, although the carbapenem MICs were lower than both the parent strain and other transformants carrying only an OXA-48−like carbapenemase (Table 4). None of the plasmids harboring *bla*_OXA-48_−like genes encoded additional carbapenemases.

### Genotypic Characterization of Isolates

We confirmed by using WGS the presence of *bla*_OXA-48_−like genes in every isolate, including the alleles *bla*_OXA-48_ (n = 7, 23%), *bla*_OXA-181_ (43%), and *bla*_OXA-232_ (33%). The gene *bla*_NDM_ was identified in 5 isolates with *bla*_OXA-232_. Nearly all isolates (93%) contained >1 ESBL gene, including *bla*_SHV-12_, *bla*_CTX-M-14b_, and *bla*_CTX-M-15_ (Table 2). We also found aminoglycoside, fluoroquinolone, sulfonamide, trimethoprim, tetracycline, chloramphenicol, macrolide, and fosfomycin resistance genes. Multilocus sequence typing of 27 *K. pneumoniae* isolates showed ST34 (n = 7), ST14 (n = 7), ST16 (n = 3), ST43 (n = 3), and ST101 (n = 3) to be most common in this collection (Table 2).

**Figure 1:**
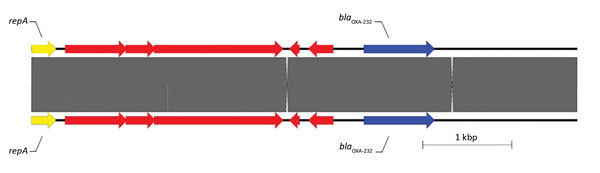
Sequence structure of 2 β-lactamase OXA-232 (*bla*_OXA-232_) plasmids tested during phenotypic and genotypic characterization of *Enterobacteriaceae* producing OXA-48–like carbapenemases, United States. Top plasmid is from isolate 11 in this study (pColKP3_DHQP1300920) (6139 bp), and bottom plasmid is from Potron et al. (*43*) (GenBank accession no. JX423831). Arrows indicate direction of transcription. Red arrows indicate other genes. OXA, oxacillinase; *repA*, COLe type replicase.

Isolates 1, 11, and 23 (carrying *bla*_OXA-181_, *bla*_OXA-232_, and *bla*_OXA-48_, respectively) were randomly chosen for Pacific Biosciences WGS in addition to Illumina WGS. Isolate 1 had 2 plasmids and encoded 20 antimicrobial drug resistance genes, including 3 chromosomal copies of the ESBL CTX-M-15; *bla*_OXA-181_ was also chromosomally located, with an upstream ΔIS*Ecp1* insertion sequence. The ΔIS*Ecp1* insertion sequence has been described elsewhere (*40**–**42*). Isolate 11 had 4 plasmids and encoded 34 antimicrobial drug resistance genes, including plasmid-mediated *bla*_CTX-M-15_ and *bla*_NDM-1_ genes. The *bla*_OXA-232_ allele in isolate 11 was found on a ColKP3 plasmid (plasmid size 6,139 bp, G + C content 52.17%); upstream of *bla*_OXA-232_, there was a ΔIS*Ecp1* insertion sequence. The sequence of this plasmid (pColKP3_DHQP1300920) has been deposited in GenBank under accession no. CP016920.1. pColKP3_DHQP1300920 was most similar to a ColKP3 plasmid previously deposited under GenBank accession no. JX423831 (100% query coverage, 99% sequence similarity) (Figure 1) (*43*).

**Figure 2:**
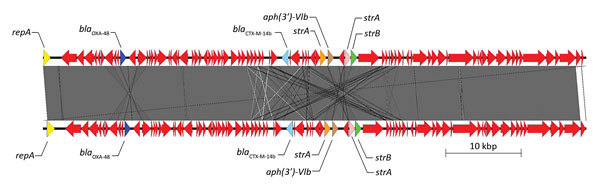
Sequence structure of 2 β-lactamase OXA-48 (*bla*_OXA-48_) plasmids tested during phenotypic and genotypic characterization of *Enterobacteriaceae* producing OXA-48–like carbapenemases, United States. Top plasmid is from isolate 23 in this study (pIncL_M_DHQP1400954) (72,093 bp), and bottom plasmid is from Chen et al. (*44*) (GenBank accession no. KP025948). Arrows indicate direction of transcription. Red arrows indicate other genes. Gray area indicates regions of homology, white lines indicate nonhomologous regions, and gray lines indicate inversions. *aph*, aminoglycoside; OXA, oxacillinase; *repA*, IncL/M type replicase; *str*, streptomycin.

Isolate 23 had 3 plasmids and encoded 16 antimicrobial drug resistance genes, including 2 ESBLs (plasmid-mediated CTX-M-14b and CTX-M-15). *bla*_OXA-48_ was present on an IncL/M plasmid (plasmid size 72,093 bp, G + C content 50.55%). This plasmid contained 89 open reading frames, including those for several antimicrobial drug resistance genes (*bla*_CTX-M-14b_, [streptomycin] *strA, strB,* and [aminoglycoside] *aph(3′)-VIb*), in addition to *bla*_OXA-48_, which appears to have been inserted into the plasmid by transposon Tn*1999.2* (GenBank accession no. JN714122). The sequence of this plasmid (pIncL_M_DHQP1400954) has been deposited in GenBank under accession no. CP016927.1. This plasmid, pIncL_M_DHQP1400954 was most similar to pOXA48-Pm (GenBank accession no. KP025948) (95% query coverage, 99% sequence similarity) (Figure 2) (*44*).

**Figure 3:**
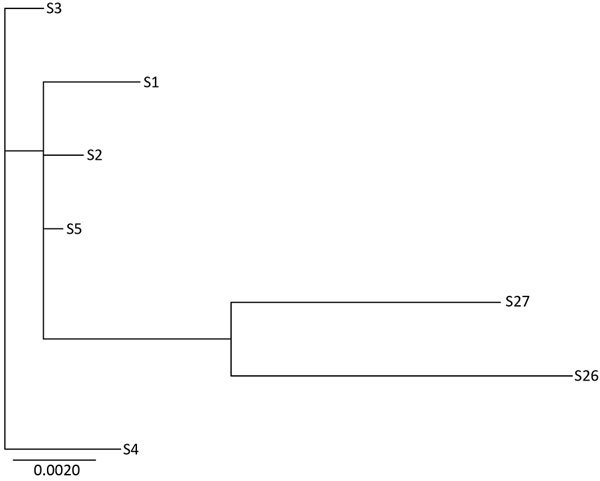
Phylogenetic tree of 7 sequence type 34 *Klebsiella pneumoniae* isolates tested during phenotypic and genotypic characterization of *Enterobacteriaceae* producing oxacillinase-48–like carbapenemases, United States. Genetic diversity ranged from 1 to 33 high-quality single-nucleotide polymorphisms that were called in an ≈5 Mb core genome, which equals ≈90% of the reference genome size (isolate 1 sequenced by using Pacific Biosciences [Menlo Park, CA, USA], technology). Scale bar indicates nucleotide substitutions per site.

We identified no SNPs when we compared Illumina and Pacific Biosciences genome sequences for the same isolate for isolates 1, 11, and 23. This finding indicates that Pacific Biosciences sequences can be used as a mapping reference. We compared Illumina sequence data for the remaining clinical isolates, which were not subjected to Pacific Biosciences sequencing, against the Pacific Biosciences genomes according to *bla*_OXA-48_−like allele. For all 10 isolates containing *bla*_OXA-232_, the gene was co-located with the ColKP3 replicon gene and a ΔIS*Ecp1* upstream insertion sequence (upstream of *bla*_OXA-232_) on an ≈6 kb contig. Pacific Biosciences sequence analysis of isolate 23 confirmed the presence of *bla*_OXA-48_ on transposon Tn*1999.2*; *bla*_OXA-48_ was found on a variant of transposon Tn*1999* in all instances. In 3 isolates (23, 28, and 29), coverage of the IS*1R* insertion sequence was similar to the overall assembly coverage suggestive of the Tn*1999.2* variant identified in isolate 23 by Pacific Biosciences sequencing. However, in 4 isolates (14, 21, 25, and 30), coverage of the IS*1R* insertion sequence was much higher than the overall assembly coverage, indicating multiple occurrences of this locus, suggestive of a different Tn*1999* variant. Of the 13 isolates containing *bla*_OXA-181_, 12 had an upstream insertion sequence ΔIS*Ecp1*. In isolate 1, which was sequenced by using Pacific Biosciences technology, *bla*_OXA-181_ was confirmed as being chromosomally located. Finally, given the geographic association of several isolates carrying *bla*_OXA-181_, we created a phylogenetic tree and SNP matrix table for the 7 *K. pneumoniae* isolates from 1 state in HHS region 9 (Table 5; Figure 3).

**Table 5:** SNP matrix for 7 *Klebsiella pneumoniae* isolates with β-lactamase oxacillinase-181–like carbapenemases from HHS region 9, United States*

Isolate no.	Isolate no.						
	26	27	4	5	1	2	3
26	0	31	33	17	32	27	28
27	31	0	27	15	28	23	26
4	33	27	0	6	13	10	8
5	17	15	6	0	5	3	1
1	32	28	13	5	0	7	9
2	27	23	10	3	7	0	6
3	28	26	8	1	9	6	0

*Genetic diversity ranged from 1 to 33 high-quality SNPs that were called in an ≈5-Mb core genome, which equals ≈90% of the reference genome size (isolate 1 sequenced by using Pacific Biosciences (Menlo Park, CA, USA) technology. *bla*, β-lactamase; HHS, Health and Human Services; SNP, single-nucleotide polymorphism.

## Discussion

The increasing prevalence of CRE in the United States poses a challenge to patients, clinicians, and public health. The diversity of carbapenemases, including the OXA-48−like enzymes reported in this study, is an ongoing diagnostic challenge to clinical microbiology laboratories because of the variety of phenotypes displayed by isolates producing different, and sometimes multiple, carbapenemases. OXA-48 has been described as the phantom menace because of its subtle phenotype in the absence of co-resistance mechanisms (*12*).

In this study, all isolates with *bla*_OXA-48_–like genes showed resistance to ertapenem, and most showed intermediate resistance or resistance to meropenem, ceftriaxone, ceftazidime, and cefepime. Three tests for carbapenemase production were performed on the isolates in this study. The modified Hodge test, performed for ertapenem or meropenem, and the mCIM showed positive results for all isolates with *bla*_OXA-48−_like genes. The Carba Nordmann–Poirel test showed positive results for 73% of all isolates, which is consistent with other studies that have shown that this test had a sensitivity of 72%–76% for OXA-48−like carbapenemase producers (*45**,**46*). All isolates in this study would be identified as CRE by the current CDC and Council of State and Territorial Epidemiologists definitions (https://www.cdc.gov/hai/organisms/cre/definition.html) (*47*).

The 10 isolates that harbored *bla*_OXA-232_ were all found on a small ColKP3 plasmid, and this association has been reported by Potron et al. (*43*). Likewise, the 7 isolates producing OXA-48 carried *bla*_OXA-48_ on a similar genetic environment to those reported (*44*,*48*,*49*). Isolate 23, which was sequenced by using Illumina and Pacific Biosciences technology, harbored *bla*_OXA-48_ on an IncL/M plasmid. The other 6 isolates, which were sequenced only by using Illumina technology, all had the IncL/M replicon gene. In addition, *bla*_OXA-48_ was always associated with a variant of transposon TN*1999*, as discerned on the basis of the copy number of IS*1R* insertion sequences (*36*). Because these IS*1R* sequences are identical and duplicated, Illumina technology often fails to assemble these as separate loci but instead produces a single locus with high coverage. Comparing coverage of the IS*1R* insertion sequence to the overall coverage of the assembly sequence enabled us to estimate the presence of the TN*1999* variant by using isolate 23 as the reference. In 12 of 13 isolates with *bla*_OXA-181_, we found an upstream ΔIS*Ecp1* element inserted upstream of the *bla*_OXA-181_ cassette. *bla*_OXA-181_ is often associated with IS*Ecp1*, which might facilitate its spread (*50*).

The transformation experiment helped to clarify our understanding of the plasmids harboring *bla*_OXA-48_−like genes. Transformation experiments were successful for each of the parent strains carrying *bla*_OXA-48_ or *bla*_OXA-232_. Carbapenem and penicillin MICs were not different between the parent and transformant, but transformant MICs were comparatively lower for cephalosporins and aminoglycosides. This finding supports the genotypic data, which indicated that ESBL genes and other β-lactamase genes did not cotransfer with the plasmid encoding *bla*_OXA-48−_like genes. One transformant (23T) did not have decreased cephalosporin MICs when compared with its parental strain, which is consistent with Pacific Biosciences sequencing of this isolate, which showed *bla*_CTX-M-14b_ to be on the same IncL/M plasmid as *bla*_OXA-48_. The unsuccessful transformation attempts of *bla*_OXA-181_–containing strains 1, 2, 26, and 27 was explained by WGS evidence that *bla*_OXA-181_ was chromosomally located in isolate 1.

We also detected a possible reservoir of isolates with *bla*_OXA-48_−like genes in the United States. Among the 13 isolates with *bla*_OXA-181_, 8 were from 1 state in HHS region 9 and contained *bla*_CTX-M-15_, *bla*_SHV-26_, and *ampH*. Seven of these isolates were *K. pneumoniae* belonging to ST34, and 5 were collected during June 2010−May 2011 (Tables 2,5; Figure 3).

This study had several limitations. The collection of isolates in this study might not be representative of all isolates with *bla*_OXA-48_−like genes in the United States. There is also a reporting bias because only isolates sent to CDC were included. CDC receives isolates as part of outbreak investigations, surveillance studies, and to confirm AST results, but there is no national requirement to submit carbapenemase-producing isolates. Thus, unusually resistant isolates are more likely to be sent to the CDC and included in this study. Also, no prevalence rates of *Enterobacteriaceae* with *bla*_OXA-48_−like genes in the United States can be inferred because there is not an evaluable denominator. In addition, almost all the isolates we studied were clinical isolates; colonizing isolates might have different phenotypic characteristics.

Another limitation is that the 10 isolates selected for the transformation experiment and the 3 isolates selected for Pacific Biosciences sequencing might not have been representative of the other isolates in this collection. Ideally, all isolates would have been sequenced by using Pacific Biosciences technology and been a part of the transformation experiment, but this testing was not performed because of limited resources. In addition, the decisions regarding which isolates to select for transformation experiments and sequencing by using Pacific Biosciences technology were made before WGS was complete. In retrospect, it would have been better to select *bla*_OXA-181_ isolates that were hypothesized to be on a plasmid for the transformation experiment; instead, chromosomal *bla*_OXA-181_ isolates were selected. Thus, the *bla*_OXA-181_ gene loci for the isolates in this study are inconclusive.

In summary, the continued increase of CRE in the United States is a major problem, and the increasing prevalence of OXA-48–like carbapenemases is also concerning. We found *Enterobacteriaceae* in the United States with *bla*_OXA-48_−like genes on similar mobile genetic elements to those described elsewhere and that displayed relatively resistant AST profiles. The first step in continued detection of CRE producing these and other carbapenemases is identifying all carbapenem resistance among *Enterobacteriaceae*, including resistance to ertapenem. Future prospective investigations are needed to determine the true prevalence of OXA-48–like carbapenemases in the United States.

Technical Appendix Additional information on phenotypic and genotypic characterization of *Enterobacteriaceae* producing oxacillinase-48–like carbapenemases, United States.
