# Correction: Vol. 24, No. 9

**DOI:** 10.3201/eid2409.C22409

**Published:** 2018-09

**Keywords:** errata, erratum, correction

Several corrections to the text were needed in Phenotypic and Genotypic Characterization of *Enterobacteriaceae* Producing Oxacillinase-48–Like Carbapenemases, United States (J.D. Lutgring et al.). The article has been corrected online (https://wwwnc.cdc.gov/eid/article/24/4/17-1377_article).

