# Supplementary material for: Phenotypic and Genotypic Characterization of Enterobacteriaceae Producing Oxacillinase-48–Like Carbapenemases, United States
Source: Emerg Infect Dis. 2018 Apr;24(4):700–9. doi: 10.3201/eid2404.171377 (PMC5875285; doi:10.3201/eid2404.171377)
Supplement: Technical Appendix — Additional information on phenotypic and genotypic characterization of Enterobacteriaceae producing oxacillinase-48–like carbapenemases, United States. [file 17-1377-Techapp-s1.pdf]

Article DOI: https://doi.org/10.3201/eid2404.171377

Phenotypic and Genotypic Characterization of *Enterobacteriaceae* Producing Oxacillinase-48–Like Carbapenemases, United States

Technical Appendix

Additional Materials and Methods

To assess clonality of plasmid sequences in our dataset, we mapped Illumina (San Diego, CA) reads from each β-lactamase oxacillinase-48–like (*bla*$_{OXA-48-}$like) isolate to the appropriate internal reference isolate sequenced by using Pacific Biosciences (Menlo Park, CA, USA) long-read technology by using the lyve-SET V1.0 pipeline (https://github.com/lskatz/lyve-SET). Single-nucleotide polymorphisms (SNPs) with 100% consensus and $\geq$5× sequencing depth were reported (*1*). We measured and visualized the number of SNPs in each pairwise comparison of a mapped isolate and the reference plasmid (Pacific Biosciences) , as well as the total number of read-covered nucleotides on each reference plasmid assembly (depth of coverage $\geq$5×), in a scatter plot by using R 3.2.3 statistical software (https://www.r-project.org/).

All Illumina contigs that harbored *bla*$_{OXA-48-}$like genes were extracted from each genome assembly and aligned against the GenBank database by means of BLASTN (https://blast.ncbi.nlm.nih.gov/Blast.cgi?PROGRAM=tblastn&PAGE_TYPE=BlastSearchto) estimate the genomic location of the *bla*$_{OXA-48-}$like gene (either plasmid or chromosome) and were also screened for plasmid replicon sequences by using the PlasmidFinder database (http://www.genomicepidemiology.org/).

A phylogenetic tree was inferred from whole-genome SNP data of 7 sequence type 34 *Klebsiella pneumoniae* isolates by means of the Lyve-Set1.1.4f pipeline (*1*). First, phage genes present in the genome sequence (Pacific Biosciences) of isolate 1 (*K. pneumoniae* sequence 34 isolate) were identified by screening against the PHAST database (*2*), and trimmed Illumina reads were subsequently mapped to unmasked regions of the reference by using SMALT (*3*).

SNPs with ≥95% consensus and ≥20× sequencing depth were reported as high-quality SNPs. To deal with possible regions of homologous recombination, leading to high-density SNP regions, we accepted 1 SNP/5 bp, a flanking distance close to the average recombination cassette length (*4*). A maximum-likelihood phylogeny was inferred from high-quality SNPs by using RAxML version 8 (*5*) and a generalized time reversible substitution model and a gamma model of rate heterogeneity.

Additional Results

The number of core plasmid SNPs between isolate 11 (pColKP3_DHQP1300920) (sequenced by using Illumina and Pacific Biosciences technology) and other isolates with *bla*$_{OXA-232}$ (sequenced by using Illumina technology) ranged from 0 to 1 and covered 5,400–6,100 bp (Technical Appendix Figure 1). Among the isolates with *bla*$_{OXA-48}$, the number of core SNPs between the plasmid sequence from reference isolate 23 (Pacific Biosciences) (pIncL_M_DHQP_1400954) and those from other OXA-48 isolates (sequenced by using Illumina technology) ranged from 371 to 430 and covered 56,000 and 62,000 bp (Technical Appendix Figure 2).

Alignments of Illumina contigs harboring *bla*$_{OXA-48–}$like run against the GenBank database showed that all contigs mapped to plasmid sequences. Alignments of Illumina contigs with *bla*$_{OXA-181}$ run against the GenBank database estimated 9 isolates to have a chromosomal location and isolates 8, 13, 20, and 24 to have a plasmid location. In 3 instances (isolates 8, 13, and 24), *bla*$_{OXA-181}$ was found on the same contig as a plasmid replicon gene (2 ColKP3 replicons and 1 IncHI1B replicon), further suggesting that *bla*$_{OXA-181}$ was located on a plasmid in those isolates.

References

1. Katz LS, Griswold T, Williams-Newkirk AJ, Wagner D, Petkau A, Sieffert C, et al. A comparative analysis of the Lyve-SET phylogenomics pipeline for genomic epidemiology of foodborne pathogens. Front Microbiol. 2017;8:375. PubMed http://dx.doi.org/10.3389/fmicb.2017.00375

2. Zhou Y, Liang Y, Lynch KH, Dennis JJ, Wishart DS. PHAST: a fast phage search tool. Nucleic Acids Res. 2011;39(suppl):W347–52. PubMed http://dx.doi.org/10.1093/nar/gkr485

3. Ponstingl H, Ning Z. SMALT: a new mapper for DNA sequencing reads. F1000 Research Posters 1:313 [cited 2018 Jan 4]. https://f1000research.com/posters/327

4. Vos M, Didelot X. A comparison of homologous recombination rates in bacteria and archaea. ISME J. 2009;3:199–208. PubMed http://dx.doi.org/10.1038/ismej.2008.93

5. Stamatakis A. RAxML version 8: a tool for phylogenetic analysis and post-analysis of large phylogenies. Bioinformatics. 2014;30:1312–3. PubMed http://dx.doi.org/10.1093/bioinformatics/btu033

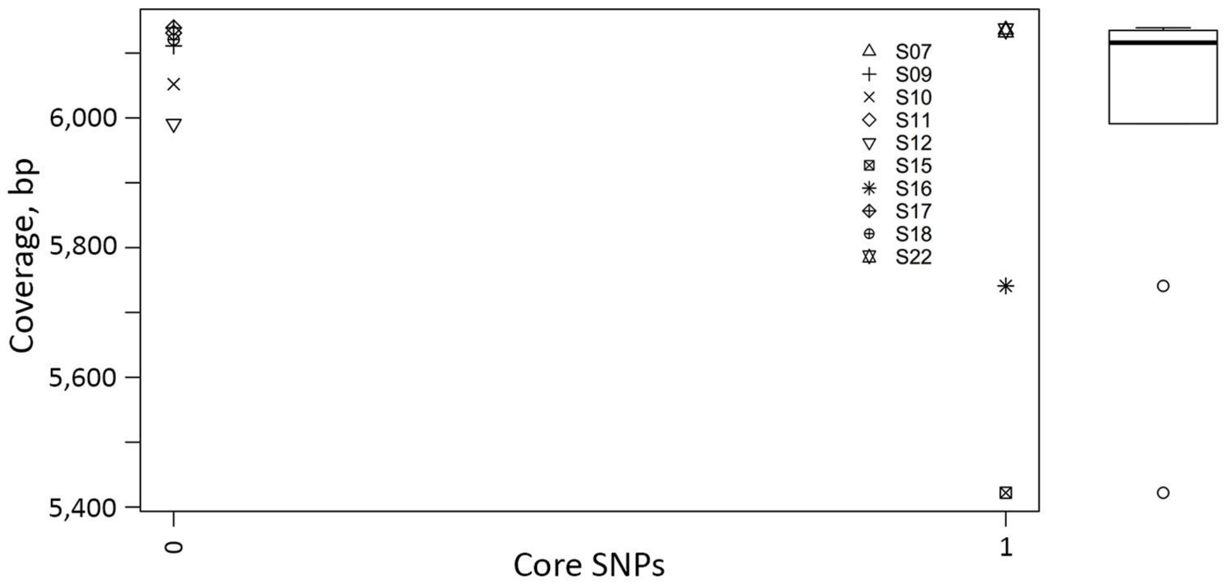

Technical Appendix Figure 1. Genetic diversity among ColKP3 plasmids that harbor β-lactamase oxacillinase-232. SNPs were identified by mapping trimmed Illumina (San Diego, CA, USA) sequencing reads to a fully closed PacBio ColKP3 plasmid assembly (isolate 11). The y-axis shows the amount of Pacific Biosciences (Menlo Park, CA, USA) reference nucleotides covered by Illumina reads of each sample (minimum of 5× coverage), and the x-axis shows the quantity of SNPs observed between the Pacific Biosciences reference and each isolate. Boxplot indicates 25th–75th interquartile ranges and relatedness among these type of oxacillinase-232–carrying plasmids in terms of shared nucleotide positions between isolates and the reference. Horizontal line in box indicates median. Outermost line (error bar) indicates 1.5× interquartile range. Only 2 isolates are considered outliers and thus more distantly related. SNP count variation ranges from 0 to 1 and indicates relatedness between isolates and the reference. SNP, single-nucleotide polymorphism.

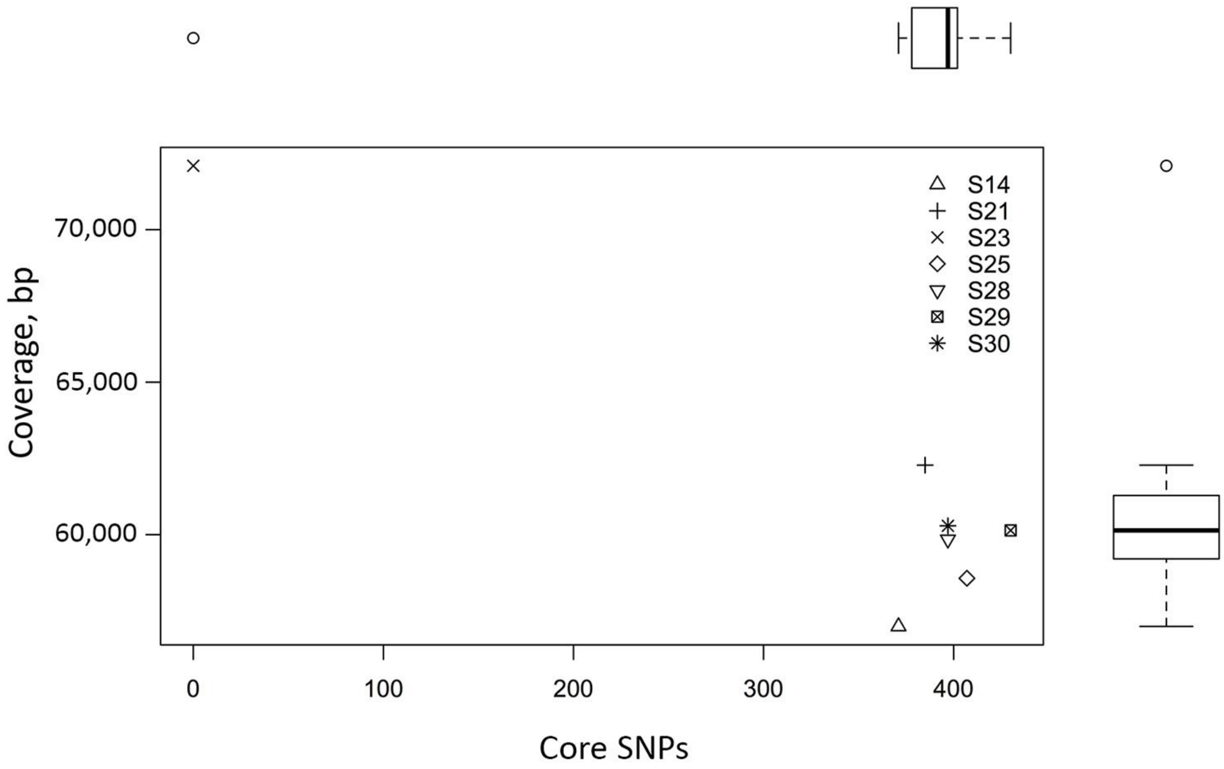

Technical Appendix Figure 2. Genetic diversity among IncL/M plasmids that harbor β-lactamase oxacillinase-48. SNPs were identified by mapping trimmed Illumina sequencing reads to a fully closed Pacific Biosciences, Menlo Park, CA, USA) IncL/M plasmid assembly (isolate 23). The y-axis shows the amount of Pacific Biosciences reference nucleotides covered by Illumina reads of each sample (minimum of 5× coverage), and the x-axis shows the quantity of SNPs observed between the Pacific Biosciences reference and each isolate. Boxplots indicate 25th–75th interquartile ranges and show that 100% coverage and 0 SNPs (indicating 100% sequence similarity) are considered features of an outlier and therefore rare in this dataset. Horizontal lines indicate medians. Outermost lines (error bars) indicate 1.5× interquartile ranges. Most IncL/M plasmids are distantly related to the Pacific Biosciences plasmid reference, indicating substantial heterogeneity of plasmids within this dataset. SNP, single-nucleotide polymorphism.
